# Investigating Learners’ Teaching Format Preferences during the COVID-19 Pandemic: An Empirical Investigation on an Emerging Market

**DOI:** 10.3390/ijerph191811563

**Published:** 2022-09-14

**Authors:** Monica Ioana Burcă-Voicu, Romana Emilia Cramarenco, Dan-Cristian Dabija

**Affiliations:** 1 Department of European Studies, Babeș-Bolyai University Cluj-Napoca, 400090 Cluj-Napoca, Romania; 2 Department of Marketing, Babeș-Bolyai University Cluj-Napoca, 400591 Cluj-Napoca, Romania

**Keywords:** emergency remote teaching (ERT), online education, e-learning platform, learners’ teaching preference, COVID-19 pandemic

## Abstract

This paper aims to measure learners’ preferences for a specific teaching format (online, hybrid, or face-to-face) based on their experience, usage, and interaction with e-learning platforms (Moodle/MS Teams), on their participation in e-learning courses delivered via online streaming platforms (Zoom), on teaching staff skills and teaching–learning abilities, as well as on the advantages and disadvantages of those forms of learning during the COVID-19 pandemic. In implementing the research question, a conceptual model was developed, which was further analyzed by means of structural equations modelling via SmartPLS 3.3.9 (SmartPLS GmbH, Boenningstedt, Germany). The data were collected via quantitative research implemented through an online questionnaire addressed to learners (students) from an emerging market during the COVID-19 pandemic. The research contributes to extending social learning theory and the social cognitive learning theory by pinpointing the learners’ preference for the online educational format and by showing how a blended learning environment in universities can be developed by fructifying the gains in terms of digital skills acquisition during the COVID-19 pandemic. The paper highlights the contribution of the online educational environment in extending the use of interactive digital tools and resources, engaging the learners, and creating the opportunity for them to become accountable for their learning experiences.

## 1. Introduction

The COVID-19 pandemic has triggered plenty of vulnerabilities in educational systems worldwide, transforming their ways of working, learning, and communicating in a sudden and dramatic manner, which challenged the limits of societies, disrupting “normality” as we knew it, and created opportunities to innovate in terms of tools and strategies [1,2,3] for the sustainable recovery of economies and societies. Throughout the COVID-19 lockdowns, emergency remote teaching (ERT) was used extensively in education worldwide. Remote teaching was initially adopted to ensure learning continuity. As the COVID-19 pandemic evolved, it became clearer that new forms of teaching and learning were needed instead of preserving superficial survival formats of education but out of necessity. Universities started to consider adaptive strategies based on innovative approaches. The opportunity to transform, innovate, and set up new forms of education had already been stressed by the literature [4,5,6,7,8]. Flexible and dynamic learning technologies have triggered the rapid increase of the online education market. It is expected that by 2025, the online education market will reach up to USD 350 billion [9], thus favoring universities’ stronger presence and commitment to online education. To ensure vital learning continuity in times of crisis for the achievement of high-performing and sustainable educational systems [10,11,12], best practices need to be made accessible and adaptive models designed and implemented [13,14,15].

The scope of this paper is to measure learners’ preferences for a specific teaching format (online, hybrid, or face-to-face) based on their experience, usage, and interaction with e-learning platforms (Moodle/MS Teams), on their participation in e-learning courses delivered via online streaming platforms (Zoom), on teaching staff skills and teaching–learning abilities, as well as on the advantages and disadvantages of those forms of learning during the COVID-19 pandemic.

The paper extends the present knowledge based on Bandura’s social learning theory [16], which focuses on human learning and behaviors, and on the social cognitive learning theory, which stresses the importance of the mutual interaction of learners with their psychosocial environment [17]. As the COVID-19 pandemic represents a new psychosocial environment, both learners and teaching staff have had to adapt to it and re-think the entire teaching–learning process. This paper explores the ways in which the peculiarities of the online environment might influence students’ learning experiences. Furthermore, the paper also adds value to the self-directed learning theory [18], which explains the way individuals improve their self-confidence, autonomy, motivation, and lifelong learning skills in educational processes. 

In implementing the research question, we develop, in the theoretical part of the paper, a conceptual model regarding learners’ preference for a specific teaching format, while in the practical part, we rely on quantitative research based on a questionnaire addressed to learners from an emerging market during the COVID-19 pandemic. The gathered data were analyzed via structural equations modelling in SmartPLS 3.3.9 (SmartPLS GmbH, Boenningstedt, Germany). The results show that the online educational environment has facilitated the extended use of interactive digital tools and resources, allowing learners to engage more with digital tools and enhancing their learning experiences.

The paper is structured as follows: Section 1 deals with the hypothesis and development of the conceptual model, while Section 2 presents the research methodology. Section 3 continues with the results and discussion of the findings, while the paper ends in Section 4 with the conclusions, consisting of theoretical contributions and managerial implications, with limitations and future research perspectives also stated. 

## 2. Hypothesis and Conceptual Model Development

### 2.1. The Social Learning Theory and the Social Cognitive Learning Theory

Proposed in the 1970s by Albert Bandura [16], the social learning theory focuses on “observing, modelling, and imitating behaviors, attitudes, and emotional reactions of others” [19]. The theory explains how both environmental and cognitive factors interact and influence human learning and behavior and is thus considered a relevant breakthrough for understanding learning processes and social behaviors [19]. Bandura’s theory also states that mediating processes occur between stimuli and responses, for instance, behavior learned from the environment through observational learning [17]. This new behavior can be acquired by observing and imitating others, as learning represents the cognitive process that takes place in a social context; it can occur purely through observation or direct instruction [20,21,22]. 

The theory of social learning was enhanced by the social cognitive learning theory, providing a framework for understanding, predicting, and changing human behavior [23] by considering the mutual influences between an individual and the physical and psychosocial environment in which he acts and his relation towards the task or behavior that must be learned [24]. These social learning theories are based on the self-regulating processes that allow and support individuals to select, refine, interpret, and transform external stimuli by relying on the self-directedness theory [18]. This theory considers that self-directed learning represents the process in which individuals take primary charge of further planning, continuing, and evaluating their own learning experiences [25]. Within the self-directed learning theory, the learning responsibility shifts from the teaching staff to the learners; hence, the proper control and the active involvement of learners within these processes is crucial for success [26,27]. 

### 2.2. Generating Learners’ Experience during the COVID-19 Pandemic

The start of the COVID-19 pandemic in early 2020 triggered a global lockdown, which affected numerous schools and universities worldwide. It is estimated that, in over 185 countries, more than 1000 million learners were affected [28]. This unprecedented context challenged universities to adapt, find new innovative solutions, and rethink the entire learning–teaching–researching process so that education could be further ensured [29]. Of course, learning deficits, as well as academic dropout rates, had to be considered, along with the necessary and rapid investments for upgrading or developing the learning IT infrastructure and sustaining efficient training programs [30]. Two years after the COVID-19 pandemic breakout, it was estimated that, at the onset of the outbreak, only 20% of universities worldwide were properly equipped with an online teaching infrastructure and with programs, with far less staff having the proper knowledge for supporting online education [31].

In the pre-COVID-19 era, the dominant format of university teaching–learning was face-to-face, with online formats being less common and mostly developed for continuous education, distant learning programs, training and/or blended learning [32]. As online education is more learner-centered, it requires more active learning and proper learner engagement compared to the classic, face-to-face teaching staff-centered learning environment), where staff control the learning environment, activate the learners, and transmit knowledge [33,34]. 

Before 2020, online education was a matter of choice when adopted by universities, and only limited research was carried out regarding online education in emergency situations, mainly regarding distance learning during SARS in Hong Kong in 2003 [35], the experience of adopting blended learning in response to the Canterbury earthquake in New Zealand in 2010 [36], or online education during the 2015–2017 protests in South Africa [37]. While the demand for distance and/or blended learning was nascent, it was considered beneficial and a future means of education [38], i.e., in “tomorrows world” [39]. Of course, implementing online education depends on the resources and infrastructure available to each university [40]. When the COVID-19 pandemic hit in early 2020, most teaching staff reacted by delivering the face-to-face content of courses and seminars via the Internet as a survival mode focused on ensuring educational continuity and on alleviating the great distress experienced by learners in lockdown [41]. As the pandemic evolved, universities started to consider strategical approaches by reconsidering their educational formats, asking teaching staff to rapidly adapt the syllabuses, teaching materials, and tools to suit online education requirements. The stress experienced by staff due to the workload, limited digital skills, and limited knowledge of digital pedagogy is also now being researched [42,43,44,45,46]. 

Universities’ commitment to invest in technological endowment must be completed by developing the teaching staff’s skills so that online education can be more efficient [41]. Teaching staff must hold cognitive and motivational competencies [47], such as pedagogical skills, content knowledge, and general pedagogical knowledge [48]. To cope with the stress in classical and online education, staff need to develop methodological competencies, soft skills, and digital competencies [49]. This means that they must learn fast and be able to use data processing and protection tools, collaborative techniques in digital environments, and know how to create digital content and/or artistic multimedia output design and how to implement online assessments, etc. [49]. In a high-quality online educational system, universities must constantly support teaching staff during the transition from classic face-to-face learning to online teaching [50,51,52,53,54]. Despite the difficulties in the rapid shift between face-to-face learning and online education, and/or vice versa, universities have made appreciable attempts to ensure that no disruption to learning and the competence flow of teaching-learning processes occurs, imposing fast adaptation to the new situation [55]. Of course, it is crucial that universities can deliver professional skills, knowledge, and competence to learners by relying on e-learning platforms and/or any means through which content can be delivered [56]. Therefore, we hypothesize that:

**Hypothesis** **1** **(H_1_).** *COVID-19 induced faculty adaptation capacity (FAC) from the pandemic has exerted a positive influence on teaching staff abilities (TSAs) accumulated during the outbreak.*

When the COVID-19 pandemic hit universities in February/March 2020, most of them had already developed ICT facilities in the form of e-learning, communication, data storage, project-based collaboration platforms, etc., which could be rapidly adapted to the new context [57]. Most of these platforms were used in an asynchronous manner, and were further developed during the COVID-19 pandemic, to be more synchronous (Zoom, Google Hangouts, Skype) and asynchronous (Google Classroom, Microsoft Teams, Moodle), with mostly open-source platforms being used [58]. Nowadays, UNESCO [31] offers updated information on those national educational platforms that were employed as a direct effect of the COVID-19 pandemic. The last two years have also brought combinations between asynchronous activities on, for example, Moodle, with synchronous activities on Zoom or MS Teams, triggering the proper adaption for universities [40,58]. Thus, we infer that:

**Hypothesis** **2** **(H_2_).** *COVID-19 induced faculty adaptation capacity (FAC) from the pandemic has a positive influence on the use of E-learning platforms (ELPs).*

To be able to teach online, teaching staff need to acquire six skills and competencies [59]: (a) pedagogical, (b) content, (c) design, (d) technological, (e) management and institutional, and (f) social and communication skills. To cope with the challenges of online teaching, staff also need to master technological pedagogical knowledge, which embeds both technical skills specific to the digital environments, as well as e-pedagogy (pedagogical and psychological aspects of online education) or digital pedagogy [60]. Using a high-performing learning machine system (LMS) like MS Teams, staff gain access to a variety of tools, enabling them to organize meetings, virtual classes, video conferencing, file storage, chat sessions, online assessments, etc. [40]. The development of such skills and competencies will support staff to efficiently use educational platforms that suit both asynchronous and synchronous activities [59]. Therefore, we formulate the hypothesis:

**Hypothesis** **3** **(H_3_).** *COVID-19 induced faculty adaptation capacity (FAC) from the pandemic positively impacts the use of video streaming platforms (VSPs).*

### 2.3. Teaching Staff Skills and the Teaching-Learning Process 

The current online teaching–learning environment has created a lot of challenges which have promoted an important change in teaching staff perspectives and the academic focus on learner-centered techniques. It implies more learner-centered approaches as opposed to the previous mainly mass-customization education process (one-size-fits-all teaching technique goal) [61]. The teaching staff abilities accumulated during the COVID-19 pandemic have shown that, to engage with and satisfy learners, it is necessary to better adapt and customize the teaching–learning process according to their needs and expectations. Therefore, teaching staff must know and heavily rely on technology-enabled learning policies and practices [59]. The online experience has highlighted the need for digital platforms capable of ensuring a favorable environment, stimulating learners’ motivation to actively engage during classes, and facilitating communication and interaction with their colleagues and educators [62]. 

Understanding learners’ needs as the basis of the online learning process represent one of the most important stages for enhancing the opportunities to adapt teaching–learning techniques [63]. Moreover, the abilities and competencies of the instructor, along with the course design and prompt feedback, will directly influence learners’ expectations and their satisfaction in the online learning process and enhance their learning outcomes [64]. There is also a positive correlation between learners’ capacity to engage in the learning process and their satisfaction with education; furthermore, the tutor’s support and academic guidance play a significant role in generating learner satisfaction [65]. 

Teaching ability for efficient, virtual-class management should enhance a collaborative digital communication environment based on digital technology [50]. To be able to promote and sustain such a process, teaching staff need to improve their methods and knowledge based on training programs and customized workshops [63]. Moreover, they should base their academic activity on constant feedback to appropriately adapt their methods and learner-centered techniques to the available digital technologies for a more engaging, high-performance, and meaningful complex learning environment [66]. In this vein, we argue that:

**Hypothesis** **4** **(H_4_).** *The teaching staff abilities (TSAs) accumulated during the COVID-19 pandemic exert a positive impact on the use of E-learning platforms (ELPs).*

Online education has been highly dependent on the existing pedagogical ability and limited knowledge of academic staff, who, in many cases, have had limited previous online teaching experience [54,67]. Teaching staff have been forced to adapt their own teaching skills, methods, materials, and techniques to suit both traditional face-to-face learning and the new digital learning experiences, focusing more on materials such as readings, videos, exercises, etc., rather than on direct interactions with learners via discussions and presentations [29]. Teaching abilities developed during the COVID-19 pandemic have succeeded in positively impacting the use of ELP. They have been mostly focused on synchronous or asynchronous mediated communication, learner-centered academic focus, and peer collaboration [50,68]. Therefore, we posit that:

**Hypothesis** **5** **(H_5_).** *The teaching staff abilities (TSAs) accumulated during the COVID-19 pandemic have a positive impact on the use of video streaming platforms (VSP).*

Online teaching has the advantage of efficiently integrating available multimedia resources, technologies, and materials, such as short clips, images, audio streaming, etc. [67]. The challenge for teaching staff is to develop their own skills for creating such resources or finding and adapting them for better use toward their academic purposes. Moreover, to attain the desired educational performance, this type of method relies on autonomous learners being able and willing to experiment and access the recommended resources by themselves [29]. The TSAs developed during the COVID-19 pandemic have generated a positive impact on video streaming platforms, depending on the complexity of the online platform [52,68]. For instance, MS Teams succeeded in creating more opportunities for applying online educational tools efficiently in comparison with other digital e-learning platforms [69]. As there is no single e-learning platform that can support a unique learning experience, the reliance on a mixture of platforms and/or tools is recommended, developing current knowledge via video streaming platforms to ensure an overall learning student experience [66]. Based on all these findings, we infer that:

**Hypothesis** **6** **(H_6_).** *The teaching staff abilities (TSAs) accumulated during the COVID-19 pandemic positively impact teaching–learning techniques (TLTs).*

The desired teaching abilities necessary for the teaching–learning processes depend on the staff’s ability to design online courses capable of transmitting relevant competencies, motivating learners to engage in learning, and enhancing the teaching–learning process [65]. For long-term knowledge retention, critical analysis of the proposed contexts, together with a problem-based learning approach, generates important benefits for learners, improving their final performance and satisfaction with the learning outcomes and helping them to better engage in knowledge acquisition [70,71,72]. Moreover, collaborative learning processes, such as teamwork, group projects, group problem-solving case studies implemented by email, mobile technology, and other forms of e-communication, are relevant content generators [7], being capable of resulting in relevant feedback for teaching staff [73]. Video materials designed for teaching purposes significantly affect the media delivery within video lectures in terms of quality issues, followed by intelligibility, pace, media diversity, and congruence [74]. Reflection topics represent a powerful teaching tool, the concepts of “reflective teaching staff” and/or the “reflective learner” having long been debated in the literature [73,75]. Nowadays, reflection not only implies an individual, intrinsic activity but also the sharing and developing of creative content, thus, exchanging valuable information within academia [8]. The online educational process offers valuable infrastructure alternatives for the dissemination of such research in the form of blogs and online journals [76]. Therefore, we formulate the following hypothesis:

**Hypothesis** **7** **(H_7_).** *The teaching staff abilities (TSAs) accumulated during the COVID-19 pandemic have generated online teaching advantages (OTAs).*

The e-learning environment allows knowledge sharing and collaboration opportunities between learners and teaching staff in the form of sharing information and ideas, uploading documents, creating and facilitating content and/or access for all, etc. [7,50,72]. Online activities have enabled learners to engage more in developing time management skills [77], thus, improving their planning and organizing abilities, and developing their skills in using a variety of educational tools available online [65], such as the use of mobile examination platforms [68]. The improvement of self-education skills is the positive result of, or complement to, e-learning [29]. The applied learning methods stimulate learners’ desires and motivations to actively engage with learning contexts and to improve self-learning practices and higher cognitive skill development [78]. Therefore, by involvement in activity-based online education, learners can become analytical thinkers and enhance their practical skills, opting for self-regulated learning practices [79]. Investigative skills are necessary, along with self-efficacy, self-learning skills, interactions, and collaborative skills, to foster e-learning [80]. Thus, we infer that:

**Hypothesis** **8** **(H_8_).** *The teaching staff abilities (TSAs) accumulated during the COVID-19 pandemic have generated online teaching disadvantages (OTDs).*

Among the disadvantages and difficulties of applying and deepening knowledge based on online-promoted teaching-learning techniques, one can also pinpoint the technical difficulties, the lack of tutor support, or the inability to solve and clarify inconsistencies that might arise during the learning process due to the reduced capacity to customize information to the specific needs of each participant in the online environment [65]. The ability to plan and organize e-learning studies is an essential tool based on efficient time management and time commitment [81]. During the COVID-19 pandemic, these aspects have led to a lot of challenges for educational stakeholders, often being considered as important barriers to the development of online educational activities due to reduced learning effectiveness and students’ motivation and affecting overall learner satisfaction and engagement [65].

The lack of interaction and communication with colleagues usually corresponds to the disadvantages identified during online educational activities, where many learners feel that online learning has created a sense of loneliness and social distancing, so they become less motivated and interested [76]. Instructional support and social presence are key motivators, along with strong learner-to-learner interactions, for the success of a good lecture [82]. Therefore, we consider that:

**Hypothesis** **9** **(H_9_).** *Teaching–learning techniques (TLTs) exhibit a positive influence on the online teaching advantages (OTAs).*

### 2.4. Challenges Encountered While Using E-Learning and Video Streaming Platforms

In comparison with the traditional learning activities performed before the COVID-19 pandemic, the new situation which forced the shift to e-learning and online classes has eased, to a certain extent, learners’ enrolment in bachelor and/or master’s study programs [83] and a moderate access perspective for learners with disabilities [84]. Furthermore, e-learning during the COVID-19 pandemic has meant that learners can more easily mix work and other professional activities with learning, thus, participating in classes even from their work office [85,86,87]. Teleworking, and the increased possibility of working from the comfort of home [87,88], have led to the better organization of daily life in terms of comfort and accessibility (timetable flexibility, freedom to organize one’s personal time, faster and more efficient communication, feedback, multitasking, etc.), saving time and reducing travelling and/or living costs [89,90]. Finally, e-learning has also impacted the work–life–learning balance, thus enhancing the feelings of psychological and medical safety [76,90]. Students can also adapt the learning pace to their own specific needs, self-discipline, and responsibility [91], and are able to improve their technological academic skills, [92] and benefit from the ease in choosing different classes while having timely and easy access to information without any logistic difficulties. In this vein, we posit that:

**Hypothesis** **10** **(H_10_).** *The online teaching advantages (OTAs) exerted a positive influence on leaners’ preferred form of teaching (SPTF) during the COVID-19 pandemic.*

Online technology should play an important part in upgrading the value of education and not be considered an instrument that will simply replace mankind and/or collaboration-based techniques. Online teaching and learning are highly dependent on both staff and learners’ knowledge of and access to existing technology [50]. Major challenges for successful e-learning activities have meant that learners must accumulate technical knowledge and digital skills, as they rely heavily on information and communication technology and internet connectivity and speed [68,72]. The potential disadvantages of using e-learning consist of the necessity of having a good command of digital skills and a proper command of the e-learning tools and platforms, the availability of e-teaching and e-learning resources, knowing how to access technical support services, being able to switch rapidly between and/or to adapt to synchronous and asynchronous activities, online assessment, etc. [63]. Therefore, we consider that:

**Hypothesis** **11** **(H_11_).** *The online teaching disadvantages (OTDs) impacted students’ preferred teaching form (SPTF) during the COVID-19 pandemic.*

Comparing e-teaching disadvantages with onsite classes, students feel less confident learning within an online setting than within a physical learning environment, as online activities often lead to distractions from their surroundings [50]. The official e-learning environment makes it even harder for students to concentrate and to clearly distinguish between school and free-time activities. Usually, entering or leaving the university campus represents a clear shift setting which delimitates the comfort of home [41]. Students’ capacity to apply accumulated knowledge by interacting with their peers and exchanging ideas is less attractive and efficient in the online educational setting and constitutes a barrier to socially meaningful interaction and collaboration compared to the face-to-face alternative [76]. 

Technological constraints, distractions, instructors’ incompetency, learners’ inefficacy, and health issues are among the most important online teaching disadvantages, diminishing students’ experience [91]. The online learning environment might also lead to health and psychosocial challenges for learners, heightening already existing learning problems. As learners need technical and digital skills and abilities, e-learning might constitute a huge challenge, especially for those who are less assertive [90]. Based on this assumption, we hypothesize that:

**Hypothesis** **12** **(H_12_).** *The use of e-learning platforms (ELPs) has had a positive influence on students’ preferred teaching form (SPTF) during the COVID-19 pandemic.*

Microsoft Teams and Moodle are frequently used e-learning platforms that easily facilitate valuable educational support for an asynchronous learning experience through proper amounts of space intended to host course materials, forums, videos, class assignments, quizzes, exams, and course calendars [90]. The COVID-19 pandemic has also triggered the use of other ways of conveying knowledge (e.g., Zoom, WhatsApp, and/or Google Classroom), but these are less attractive in terms of asynchronous learning opportunities [50,68]. 

Students are often confronted with the simultaneous use of several online e-learning platforms preferred by different teaching staff. Coping with all of these e-learning platforms might be difficult at first and requires logistical adjustments. To ease transitions and to have better control and overview of the content offered to learners, several universities have developed or relied on a single online platform for the entire university [67]. Online lectures, virtual learning environments, online e-learning platforms, oral online presentations, online tests and exams, the intensive use of technologies, etc., have also led to “strong negative emotions”, with students encountering a “lack of empathy, frustration, fear, aversion, and anxiety as sometimes it is quite stressful to take online exams and/or fulfil online assessments” [90]. In this vein, we posit that:

**Hypothesis** **13** **(H_13_).** *The use of video streaming platforms (VSPs) has had a positive influence on students’ preferred teaching form (SPTF) during the COVID-19 pandemic.*

**Figure 1:**
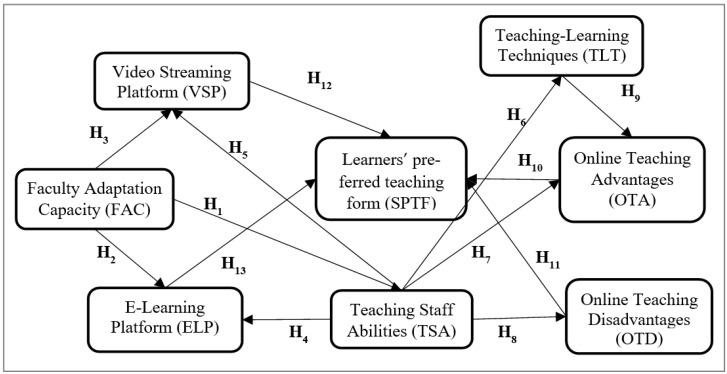
The conceptual model: Learners’ preferred teaching form during the COVID-19 pandemic. Source: own development.

Based on theoretical developments, the authors propose the conceptual model rendered in Figure 1. This model analyzes the influence of students’ preferences for a specific learning format (online, hybrid, or face-to-face) as a result of their experiences of using e-learning platforms (Moodle/Teams), participation in courses delivered via online streaming platforms (ZOOM), the teaching ability and teaching–learning techniques used by teaching staff and the advantages and disadvantages of these learning formats during the COVID-19 pandemic (see Figure 1).

## 3. Research Methodology

### 3.1. Research Design, Sampling, and Data Collection

The research is based on an empirical analysis, implemented through a survey based on an online questionnaire distributed to learners via social media platforms during the COVID-19 pandemic. We considered convenience sampling, as the goal was to attract as many students as possible. The research was carried out within the most relevant university in Romania, according to international ranking systems (Babeș-Bolyai University). Since 2020, the university has been a member of the international network GUILD, ranking first in the country for research, number of students and staff, visibility, international recognition, etc. [93].

For a better understanding of the impact of online teaching and learning processes, we carried out a quantitative analysis using a questionnaire as the main instrument for collecting the necessary data. The questionnaire was launched online via Google Forms in late 2021. The sample consists of undergraduate students enrolled in different semesters who were confronted with both on-site and online learning. From about 1200 enrolled students, we obtained a response rate of 16%, with 88% of these enrolled in BA programs and 12% in MA programs. The ratio of male to female students was 69.5% to 30.5%, respectively. As far as our sample age structure is concerned, most of the respondents fell into the age category between 18 and 22 (72.7%), while the other two age segments were almost equally represented between those over 22 and 25 years of age. A total of 73.9% students originated from urban areas, while the remaining 26.1% came from rural areas. The invitation to participate in the research was launched by the teaching staff who interacted with the students, several reminders being sent during the collection period.

### 3.2. The Evaluation of the Measurement Models

The relations between the constructs presented in Figure 1 were analyzed with the help of structural equations modelling in SmartPLS 3.3.9 (SmartPLS GmbH, Boenningstedt, Germany). All the reflective constructs were checked regarding validity and internal consistency, item loadings, average variance extracted (AVE), reliability indicators, and discriminant validity being reproduced and calculated in Table 1. As can be seen, all loadings are above the minimum threshold of 0.70, which allows us to state that the calculations used measured those items that have convergence validity [94]. The minimum and maximum values of the item loadings vary between 0.708 and 0.910, thus, are above the recommended minimum of 0.7. Reliability was tested using Cronbach’s α, which must have values above 0.7 according to the literature specifications [95]. 

The relations between the constructs presented in Figure 1 were analyzed with the help of structural equations modelling in SmartPLS 3.0. All the reflective constructs were checked regarding validity and internal consistency, item loadings, average variance extracted (AVE), reliability indicators.

As seen in Table 2, all the considered constructs meet this criterion demand, with the Cronbach Alpha result being higher than the threshold of 0.7. On the other hand, the average variance extracted for the considered constructs exceeds the requirement threshold of 0.5, indicating that the analysis model is correct [107], respectively, and that all constructs have convergent validity. The composite reliability (CR) for the constructs of the model in Figure 1 is above the threshold of 0.7, which indicates the construct’s reliability [94]. The testing of discriminant validity for each dimension consisted in applying the Fornell–Larcker criterion (Table 2) and Heterotrait–Monotrait criterion (Table 3). Thus, for the Fornell–Larcker criterion, for each latent variable the average variance extracted must be higher than the correlation coefficient between the competent and all the distinct variables [94]. 

The level of collinearity of the items in the measurement model for the dataset was further addressed. The VIF value of all indicators is below 5, which is considered the threshold in the collinearity analyses [108]. The highest value is 3.533 (OTA1 item) for the dataset, indicating there is no multicollinearity. Next, a bootstrap procedure was applied to test the hypotheses and the relationships between the latent variables. Eleven hypotheses were accepted with a significant, positive relationship based on t-statistics. 

### 3.3. The Evaluation of the Structural Models

To assess the structural model, we have also analyzed the collinearity of the constructs. The highest VIF value of the inner model is 1.528 (FAC → ELP), thus below the threshold value, indicating that there is no multicollinearity between constructs. The goodness of fit of the saturated model is also acceptable. The square root mean residual (SRMR) has a value of SRMR = 0.063 which fulfils the recommended criteria < 0.08, while the NFI is 0.961 [108]. 

Online teaching advantage, online teaching disadvantage, video streaming platform use during the COVID-19 pandemic and e-learning platform use during the COVID-19 pandemic explain 21.6% of the variance of students’ preferred teaching form (R^2^ = 0.216), while teaching learning techniques used during the COVID-19 pandemic and teaching staff abilities during the COVID-19 pandemic explain 55.8% in the variance of online teaching advantage (R^2^ = 0.588). Teaching staff abilities during the COVID-19 pandemic explain 47.5% of the variance of teaching–learning techniques used during the COVID-19 pandemic (R^2^ = 0.475). 

**Figure 2:**
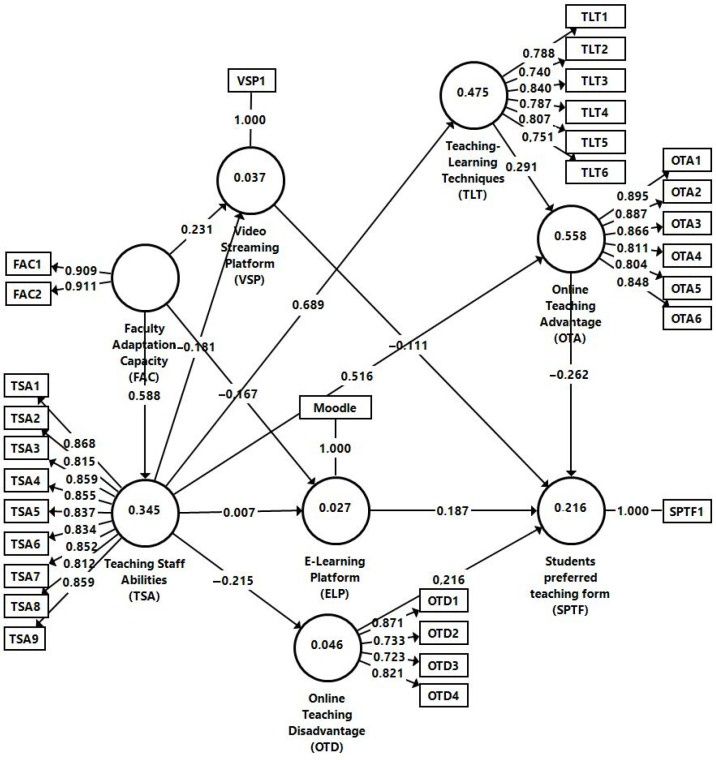
Structural model. Source: own computation in SmartPLS.

3.7% in the variance of video streaming platform use during the COVID-19 pandemic is explained by the faculty adaptation capacity to the COVID-19 pandemic and teaching staff abilities during the COVID-19 pandemic (R^2^ = 0.037), while 2.7% in the variance of e-learning platform use during the COVID-19 pandemic is explained by the faculty adaptation capacity to the COVID-19 pandemic and the teaching staff abilities gained during the COVID-19 pandemic (R^2^ = 0.027) and 4.6% in the variance of online teaching disadvantage is explained by the teaching staff abilities (R^2^ = 0.046), 34.5% in the variance of teaching staff abilities during the COVID-19 pandemic is explained by the faculty adaptation capacity to the COVID-19 pandemic (see Figure 2).

## 4. Results and Discussion

Table 4 contains the results of the hypothesis testing. H_1_ inferred that the COVID-19-induced faculty adaptation capacity from the pandemic exerts a positive influence on the teaching staff abilities accumulated during the COVID-19 pandemic. The results (β = 0.588; *T*-value = 8.753 and *p* < 0.001) prove the strong positive and significant meaning of this relationship, and therefore H_1_ is validated. Our results are supported by the literature [50,109], which stresses the relevance of faculty adaptation and support that is provided to teaching staff, especially technical back-up for efficient use of e-platforms. 

H_2_ assumed that the COVID-19-induced faculty adaptation capacity from the pandemic has had a positive influence on the use of e-learning platforms. The results (β = −0.167; *T*-value = 1.439, and *p* = 0.151^n.s.^) show that there is no meaningful and significant relationship between these two dimensions, and therefore H_2_ is rejected. Our results are distinct from several studies in that we revealed that universities which ensured access to functional e-platforms (even customized platforms) contributed to an increase in the use of e-platforms during the pandemic [29,50]. 

H_3_ presumed that the COVID-19-induced faculty adaptation capacity from the pandemic has positively impacted the use of video streaming platforms. The results (β = 0.231; *T*-value = 2.548 and *p* = 0.011) prove that the relationship is quite strong and significant, and therefore H_3_ is confirmed. Our results are consistent with the literature [67], which stressed the relevance of free streaming platforms (specifically ZOOM) promoted by higher education institutions during the initial stage of the pandemic. Zoom’s resemblance to the social media apps already used by learners facilitated the transition to an online environment. The large use of the ZOOM platform in the early stages of the pandemic is considered to have been quite friendly and easy to use, ensuring connectivity. Later, its educational facilities were discovered by the users or developed by the provider. Familiarity with using the platform led to the lexicalization of the term zooming [76].

H_4_ hypothesized that the teaching staff abilities accumulated during the COVID-19 pandemic exerted a positive impact on the use of e-learning platforms. The results (β = 0.007; *T*-value = 0.059 and *p* = 0.953^n.s.^) pinpoint that there is no relationship between the two constructs, and thus, H_4_ is not confirmed based on the empirical data. A potential explanation for this outcome resides in the fact that the immediate shift to the online environment was caused by pandemic safety measures taken at a global level and the clear need to ensure continuity [67,110]. Nevertheless, the literature [108] also pinpointed the contribution of teaching staff skills and perseverance to the students’ use of e-platforms. 

H_5_ started from the premise that the teaching staff abilities accumulated during the COVID-19 pandemic have had a positive impact on the use of video streaming platforms. The results (β = −0.181; *T*-value = 1.811 and *p* = 0.058) show a moderately strong but negative and partially significant relation between the constructs; hence, H_5_ is also partially confirmed (see Table 4). The literature review underlines the fact that, in relation to the online learning environment, instructional video plays a significant role [109]. Our research seeks to complement other endeavors by naming the use of online teaching–learning platforms like Zoom, Google Meet, Facebook, and YouTube [67] as adaptive instruments to hand but highly dependable on the teaching staff’s ability to integrate and adapt the available video streaming platforms into online lectures. 

H_6_ presumed that the teaching staff abilities accumulated during the COVID-19 pandemic had positively impacted teaching–learning techniques. The results (β = 0.689; *T*-value = 13.176 and *p* < 0.001) highlight a strong and positive influence between the teaching staff abilities accumulated during the COVID-19 pandemic and the teaching–learning techniques applied during the COVID-19 pandemic, so H_6_ is confirmed. The teaching abilities accumulated during the pandemic period have positively impacted the use of video streaming platforms. The greatest importance is allocated to the way in which teaching staff ability (to design online courses) will lead to competency development while also motivating learners [65]. Video materials and video lectures created for the online learning environment and educational purposes impact educational quality issues in terms of the way in which teaching staff control the adequate delivery of media resources to enhance the overall learning experience [74]. 

H_7_ presumed that the teaching staff abilities accumulated during the COVID-19 pandemic generated online teaching advantages. The results (β = 0.516; *T*-value = 5.644 and *p* < 0.001) largely support this assumption, proving that the teaching staff abilities accumulated during the COVID-19 pandemic have indeed generated online teaching advantages, thus confirming H_7_. The way in which teaching staff abilities succeeded in increasing the efficiency of the online learning process determines a set of recognized online advantages [111,112], represented by increased flexibility and access to learning opportunities, easier contact with experts, greater exposure to various educational environments, easier access to a wide range of courses, and better opportunities for joining student communities [52].

H_8_ was based on the premise that the teaching staff abilities accumulated during the COVID-19 pandemic also generated online teaching disadvantages. The results (β = −0.215; *T*-value = 2.462 and *p* = 0.014) confirm the relatively strong but negative influence of the teaching staff abilities accumulated during the COVID-19 pandemic on online teaching disadvantages, partially confirming H_8_. Thus, we may consider that teaching staff ability does not generate online teaching disadvantages. In other words, the teaching staff made considerable efforts to adapt to the online formats and to deliver an accessible teaching–learning process in a smooth manner for their students. Our results are consistent with other studies that revealed the tremendous effort of teaching staff to adapt fast and deliver valuable e-content to their students [55,113]. 

H_9_ studied the influence of teaching–learning techniques on online teaching advantages. The results obtained (β = 0.291; *T*-value = 2.926 and *p* < 0.005) showed a relatively strong and significant positive impact of teaching–learning techniques on online teaching advantages. So, H_9_ is also confirmed. Similar research [47] has shown the contribution of teaching skills in the online format to students’ perceived advantage in the learning process. 

H_10_ analyzed whether the online teaching advantages exerted a positive influence on students’ preferred teaching form during the COVID-19 pandemic. In this case, we noticed that the results (β = −0.262; *T*-value = 3.152 and *p* < 0.005) showed a relatively strong and significant but negative influence of online teaching advantages on students’ preferred teaching form during the COVID-19 pandemic, partially confirming H_10_. Therefore, despite the major advantages of online education (flexibility, self-learning development, improvement of technical skills, and inter-connectivity to mention a few), students are aware that knowledge acquisition and skill development are highly dependent on their interaction with teaching staff in face-to-face learning formats. Other studies [50,76] came to similar conclusions, pinpointing the shortcomings of online learning (despite accessibility, in-person communication, and inter-personal communication being performed to a higher level in face-to-face learning formats).

On the other hand, H_11_ investigated the impact of teaching disadvantages on students’ preferred teaching form during the COVID-19 pandemic. The results (β = 0.216; *T*-value = 3.352 and *p* < 0.001) show a moderate positive influence, and a strongly significant influence between the two constructs, and therefore, H_11_ is also confirmed. Online teaching disadvantages [113], such as internet browsing issues, computer compatibility, technical issues [52], lack of interaction, and training [114], strongly influenced students’ preferred teaching format. H_12_ started from the premise that the use of e-learning platforms has a positive influence on students’ preferred teaching form during the COVID-19 pandemic. The results (β = 0.187; *T*-value = 2.548 and *p* < 0.05) prove a moderate positive and moderate significant meaning to this relationship; therefore, H_12_ is confirmed. Previous research [32,50] has found a strong significant influence between e-learning platforms and students’ preferred teaching forms.

H_13_ assumed that the use of video streaming platforms positively impacted students’ preferred teaching form during the COVID-19 pandemic. The results (β = −0.111; *T*-value = 1.601 and *p* = 0.110^n.s.^) pinpoint that there is no relation between the two dimensions, and therefore, H_13_ is not confirmed. This outcome might be explained by the fact that, in the early stages of the COVID-19 pandemic, these platforms were only hosting courses and seminars, preserving the features of face-to-face format [61] in a needs-based-use approach [67]. Our results are contrary to previous studies, revealing students’ manifest preference for online formats due to the ease of video streaming platforms access [115]. 

The need to provide a consistent theoretical background should incorporate tactics concentrating mostly on delivering content, knowledge, skills, valuable information, and supportive tools for students to connect with empathic and resilient teaching staff, which may lead to long-term returns [116,117]. Among the used techniques, one can highlight play-based learning, breaking down learning content into smaller work packages, creating a safe (e-)learning environment, fostering relationships, furthering reflective reasoning and analysis, offering appropriate praise, guiding students to discover new knowledge, helping them to better understand their personality and strengths, providing appropriate challenges, configuring clear learning objectives that target explicit skills, using a hands-on approach, etc. [118].

Short-term adaptive actions tended to initially concentrate mostly on information delivery (the focus was on ensuring learning continuity by providing a variety of online learning materials for students). Initially, the teaching staff were mostly evaluating the content from a quantitative perspective to make up for the lack of direct interaction instead of allocating more time to test and implement efficient modes of information delivery. The quality in delivering correct value-added educational resources for students resides in understanding what the students really need in the newly created environment. This includes connecting available knowledge, arguing, analyzing the implications, being able to think creatively and adapt to changes, being proactive and thinking of outcomes, etc. [29]. The new online environment has created the opportunity for more multi-modal diverse settings that allow teaching staff to choose from a broad variety of available alternatives related to technological aspects, online platforms, informational sources and resources, or combined teaching methods. This reality has become the new normality which is here to stay “due to enhanced infrastructure and developed skill sets that allow people to move across different delivery systems” ([119], p. 5).

Previous research on students’ perceptions of face-to-face, online, or hybrid/blended learning formats in a non-COVID related context reveals, for instance, a general positive perception of online education [120,121] regardless of if students have been previously exposed to online learning [122] or not [111]. Positive perceptions of face-to-face versus online learning have also been pinpointed in Portugal [123], Bangladesh [124], Ukraine [125] and Romania [126,127].

## 5. Conclusions

From a theoretical perspective, our research extends the social learning theory developed by Bandura [16], which stressed the role of the learning process induced by human behavior. We show that the contribution of the psychosocial environment enhances individual learning processes. Our conceptual model reveals the overall contribution of the online educational environment, which has facilitated the extended use of interactive digital tools and resources, engaged learners, and created an opportunity for them to become accountable for their learning experience. Of course, more research on national response measures, adopted models, and prospective strategies are needed. Our contribution should be understood as an analysis of students’ preference for online education as a prerequisite for an efficiently reformed educational system in Romania.

From policymakers’ perspective, the paper pinpoints several possible benchmarks that could be used further by universities to enhance learners’ satisfaction and increase their participation in online and hybrid classes. The contemporary student is keen to use different online tools which should be further used by teaching staff in both face-to-face and hybrid education. Adapting to the COVID-19 pandemic is, and remains, a challenge for universities, as large numbers of students make full face-to-face education less possible. Furthermore, relying on online tools remains a must for universities in the 21^st^ century. 

Among the limitations of the research, we pinpoint the small sample size, as well as the fact that the study was only implemented in one faculty of the university. Future research should thus consider approaching more students and/or more faculties, if possible, from different fields, such as the natural sciences, humanities, social sciences, engineering, etc. Another limitation could be the fact that the research was only carried out in one university. Future research could increase the number of universities, also allowing for cross-national and/or cross-cultural comparisons. More benchmarks in adapting to the COVID-19 situation are necessary to find suitable solutions for future possible disruptions to teaching–learning processes. Some of the already available best practices could be transposed within conflict situations and/or zones, thus allowing learners to be further engaged in teaching–learning processes.

## Figures and Tables

**Table 1:** Constructs and items.

Item	Measure	Loading
Faculty Adaptation Capacity (FAC) adapted after [50,51,96].		
FAC1	The faculty’s capacity to adapt to the new context and to deliver knowledge in the new context is high	0.909
FAC2	Students’ satisfaction related to the use of educational platforms is high	0.911
Teaching Staff Abilities (TSA) adapted after [29,50,52,59,89,97,98].		
TSA1	The teaching staff’s high capacity to adapt to online teaching is important in online education	0.868
TSA2	The teaching staff developed abilities to set deadlines are important in online education	0.815
TSA3	The teaching staff’s clarity in designing tasks is important in online education	0.859
TSA4	The teaching staff developed skills to use a variety of online teaching tools are important in online education	0.855
TSA5	The teaching staff’s ability to provide feedback and assessments is important in online education	0.837
TSA6	The teaching staff’s technical skills are important in online education	0.834
TSA7	The balance between theoretical and practical components in the online teaching process is relevant	0.852
TSA8	The teaching staff’s ability to arouse attention and to maintain interest is important in online education	0.812
TSA9	The teaching staff’s ability to support students in the learning process is important in online education	0.859
Video Streaming Platform (VSP) adapted after [66,99,100].		
VSP1	The most used video streaming platform was Microsoft Teams	1.000
e-Learning Platform (ELP) adapted after [99,100,101]		
ELP1	The most utilized e-learning platform was Moodle	1.000
Teaching-Learning Techniques (TLT) adapted after [65,70,73,74].		
TLT1	The individual seminar activity generated the greatest efficiency for the learning process	0.788
TLT2	The critical analysis of some proposed contexts generated the greatest efficiency for the learning process	0.740
TLT3	Working in teams has generated the greatest efficiency for the learning process	0.840
TLT4	Video materials have generated the greatest efficiency for the learning process	0.787
TLT5	Case studies have generated the greatest efficiency for the learning process	0.807
TLT6	Reflection topics generated the greatest efficiency for the learning process	0.751
Online Teaching Advantage (OTA) adapted after [50,64,65,77,80].		
OTA1	Access to information available in the virtual environment as a complement to the educational process, an advantage identified in relation to online teaching	0.895
OTA2	Ability to plan and organize as a complement to the educational process, an advantage identified in relation to online teaching	0.887
OTA3	Development of skills to use the educational tools available online as a complement to the educational process, an advantage identified in relation to online teaching	0.866
OTA4	Time efficiency as a complement to the educational process, an advantage identified in relation to online teaching	0.811
OTA5	Feedback received during the semester as a complement to the educational process, an advantage identified in relation to the online teaching activity	0.804
OTA6	Improving self-education skills as a complement to the educational process, an advantage identified in relation to online teaching	0.848
Online Teaching Disadvantage (OTD) adapted after [65,76,81,82,102,103,104,105].		
OTD1	Difficulties regarding the ability to apply and deepen the knowledge acquired during the studies carried out correspond to the disadvantages identified during the development of the online educational activity	0.871
OTD2	The ability to plan and organize corresponds to the disadvantages identified during the development of online educational activity	0.733
OTD3	Interaction and communication with colleagues correspond to the disadvantages identified during the online educational activity	0.723
OTD4	Solving and clarifying inconsistencies corresponds to the disadvantages identified during the online educational activity	0.821
Students preferred teaching form (SPTF) adapted after [106].		
STF1	To what extent can the decision to continue studies in an online teaching-learning system be negatively influenced?	1.000

Source: own development.

**Table 2:** Discriminant validity analyses (Fornell–Larcker criterion).

Cronbach Alpha	AVE	CR	Con-Struct	ELP	FAC	OTA	OTD	SPTF	TSA	TLT	VSP
1.000	1.000	1.000	ELP	1.000							
0.792	0.828	0.906	FAC	−0.163	0.910						
0.924	0.727	0.941	OTA	−0.199	0.675	0.852					
0.819	0.624	0.868	OTD	−0.019	−0.355	−0.254	0.790				
1.000	1.000	1.000	SPTF	0.249	−0.335	−0.353	0.284	1.000			
0.949	0.712	0.957	TSA	−0.091	0.588	0.716	−0.215	−0.288	0.844		
0.876	0.618	0.907	TLT	−0.168	0.538	0.647	−0.141	−0.209	0.689	0.786	
1.000	1.000	1.000	VSP	−0.123	0.125	−0.006	−0.049	−0.143	−0.045	−0.060	1.000

Note: ELP: E-Learning Platform use during the COVID-19 pandemic; FAC: Faculty Adaptation Capacity to the COVID-19 pandemic; OTA: Online Teaching Advantage; OTD: Online Teaching Disadvantage; SPTF: Students Preferred Teaching Form; TSA: Teaching Staff Abilities during the COVID-19 pandemic; TLT: Teaching Learning Techniques used during the COVID-19 pandemic; VSP: Video Streaming Platform use during the COVID-19 pandemic. Cronbach Alpha > 0.7; Average Variance Extracted (AVE) > 0.5; Composite Reliability (CR) > 0.7.

**Table 3:** Discriminant validity analyses (Heterotrait–Monotrait criterion).

Construct	ELP	FAC	OTA	OTD	SPTF	TSA	TLT	VSP
ELP								
FAC	0.183							
OTA	0.208	0.788						
OTD	0.085	0.416	0.253					
SPTF	0.249	0.377	0.366	0.265				
TSA	0.098	0.675	0.761	0.210	0.295			
TLT	0.179	0.647	0.719	0.159	0.224	0.753		
VSP	0.123	0.141	0.030	0.051	0.143	0.066	0.097	

Note: ELP: E-Learning Platform use during the COVID-19 pandemic; FAC: Faculty Adaptation Capacity to the COVID-19 pandemic; OTA: Online Teaching Advantage; OTD: Online Teaching Disadvantage; SPTF: Students Preferred Teaching Form; TSA: Teaching Staff Abilities during the COVID-19 pandemic; TLT: Teaching Learning Techniques used during the COVID-19 pandemic; VSP: Video Streaming Platform use during the COVID-19 pandemic. Cronbach Alpha > 0.7; Average Variance Extracted (AVE) > 0.5; Composite Reliability (CR) > 0.7.

**Table 4:** The path coefficients of the structural equation model.

Paths	PathCoefficients	Standard Deviation	*T*-Value	CI ^1^	*p*-Value	Hypotheses
FAC → TSA	0.588	0.067	8.753	0.434~0.711	0.000 ***	H_1_-Confirmed
FAC → ELP	−0.167	0.116	1.439	−0.376~0.096	0.151 ^n.s.^	H_2_-Not confirmed
FAC → VSP	0.231	0.091	2.548	0.048~0.391	0.011 **	H_3_-Confirmed
TSA → ELP	0.007	0.122	0.059	−0.223~0.233	0.953 ^n.s.^	H_4_-Not confirmed
TSA → VSP	−0.181	0.100	1.811	−0.359~0.028	0.058 *	H_5_-Partially confirmed
TSA → TLT	0.689	0.052	13.176	0.572~0.780	0.000 ***	H_6_-Confirmed
TSA → OTA	0.516	0.091	5.644	0.341~0.687	0.000 ***	H_7_-Confirmed
TSA → OTD	−0.215	0.087	2.462	−0.356~0.015	0.014 **	H_8_-Partially confirmed
TLT → OTA	0.291	0.100	2.926	0.097~0.472	0.004 **	H_9_-Confirmed
OTA → SPTF	−0.262	0.083	3.152	−0.419~−0.099	0.002 **	H_10_-Partially confirmed
OTD → SPTF	0.216	0.064	3.352	0.057~0.323	0.001 ***	H_11_-Confirmed
ELP → SPTF	0.187	0.073	2.548	0.045~0.317	0.011 **	H_12_-Confirmed
VSP → SPTF	−0.111	0.069	1.601	−0.250~0.026	0.110 ^n.s.^	H_13_-Not confirmed

Note: * *p* < 0.1; ** *p* < 0.05; *** *p* < 0.001; ^n.s.^ not significant. ELP: E-Learning Platform use during the COVID-19 pandemic; FAC: Faculty Adaptation Capacity to the COVID-19 pandemic; OTA: Online Teaching Advantage; OTD: Online Teaching Disadvantage; SPTF: Students Preferred Teaching Form; TSA: Teaching Staff Abilities during the COVID-19 pandemic; TLT: Teaching Learning Techniques used during the COVID-19 pandemic; VSP: Video Streaming Platform use during the COVID-19 pandemic. ^1^ CI=Confidence Interval (2.5–97.5%).

## Data Availability

Not applicable.
